# Plasma metabolomics reveals membrane lipids, aspartate/asparagine and nucleotide metabolism pathway differences associated with chloroquine resistance in *Plasmodium vivax* malaria

**DOI:** 10.1371/journal.pone.0182819

**Published:** 2017-08-16

**Authors:** Karan Uppal, Jorge L. Salinas, Wuelton M. Monteiro, Fernando Val, Regina J. Cordy, Ken Liu, Gisely C. Melo, Andre M. Siqueira, Belisa Magalhaes, Mary R. Galinski, Marcus V. G. Lacerda, Dean P. Jones

**Affiliations:** 1 Clinical Biomarkers Laboratory, Division of Pulmonary Medicine, Department of Medicine, Emory University, Atlanta, Georgia, United States of America; 2 Malaria Host–Pathogen Interaction Center, Atlanta, Georgia, United States of America; 3 International Center for Malaria Research, Education and Development, Emory Vaccine Center, Yerkes National Primate Research Center, Emory University, 954 Gatewood Road, Atlanta, Georgia, United States of America; 4 Rollins School of Public Health, Emory University, Atlanta, Georgia, United States of America; 5 Universidade do Estado do Amazonas, Manaus, Amazonas, Brazil; 6 Fundação de Medicina Tropical Dr. Heitor Vieira Dourado, Manaus, Amazonas, Brazil; 7 Instituto Nacional de Infectologia Evandro Chagas (FIOCRUZ), Rio de Janeiro, Rio de Janeiro, Brazil; 8 Instituto Leônidas & Maria Deane (FIOCRUZ), Manaus, Amazonas, Brazil; Institute of Tropical Medicine (NEKKEN), JAPAN

## Abstract

**Background:**

Chloroquine (CQ) is the main anti-schizontocidal drug used in the treatment of uncomplicated malaria caused by *Plasmodium vivax*. Chloroquine resistant *P*. *vivax* (PvCR) malaria in the Western Pacific region, Asia and in the Americas indicates a need for biomarkers of resistance to improve therapy and enhance understanding of the mechanisms associated with PvCR. In this study, we compared plasma metabolic profiles of *P*. *vivax* malaria patients with PvCR and chloroquine sensitive parasites before treatment to identify potential molecular markers of chloroquine resistance.

**Methods:**

An untargeted high-resolution metabolomics analysis was performed on plasma samples collected in a malaria clinic in Manaus, Brazil. Male and female patients with *Plasmodium vivax* were included (n = 46); samples were collected before CQ treatment and followed for 28 days to determine PvCR, defined as the recurrence of parasitemia with detectable plasma concentrations of CQ ≥100 ng/dL. Differentially expressed metabolic features between CQ-Resistant (CQ-R) and CQ-Sensitive (CQ-S) patients were identified using partial least squares discriminant analysis and linear regression after adjusting for covariates and multiple testing correction. Pathway enrichment analysis was performed using Mummichog.

**Results:**

Linear regression and PLS-DA methods yielded 69 discriminatory features between CQ-R and CQ-S groups, with 10-fold cross-validation classification accuracy of 89.6% using a SVM classifier. Pathway enrichment analysis showed significant enrichment (*p*<0.05) of glycerophospholipid metabolism, glycosphingolipid metabolism, aspartate and asparagine metabolism, purine and pyrimidine metabolism, and xenobiotics metabolism. Glycerophosphocholines levels were significantly lower in the CQ-R group as compared to CQ-S patients and also to independent control samples.

**Conclusions:**

The results show differences in lipid, amino acids, and nucleotide metabolism pathways in the plasma of CQ-R versus CQ-S patients prior to antimalarial treatment. Metabolomics phenotyping of *P*. *vivax* samples from patients with well-defined clinical CQ-resistance is promising for the development of new tools to understand the biological process and to identify potential biomarkers of PvCR.

## Introduction

Malaria is a global health infectious disease problem with almost half of the world’s population at risk. Among the *Plasmodium* species causing malaria, *P*. *vivax* is the leading cause of malaria in extensive areas of the world [1], with more than 8.5 million cases estimated in 2015 [2]. Most common manifestations of severe vivax malaria include severe anemia and respiratory distress, and these are particularly associated with young age [3–5]. The decline in malaria in the Americas since the 1990’s continues to fuel hope for its eventual elimination [6]. However, emerging resistance to antimalarial treatment poses a threat to such efforts. Chloroquine (CQ) is the main drug used in the treatment of uncomplicated vivax malaria. CQ accumulates in the digestive vacuole, an acidic compartment, of the parasite [7]. The high intravacuolar concentration of CQ is proposed to inhibit heme biocrystallization, thus leading to heme build up in toxic levels and inhibition of various processes in the parasite cell [8, 9]. Various proteins and lipids have been implicated in pigment biocrystallization, but their precise role and the effect of drugs in the process remains to be elucidated [10]. Furthermore, CQ is active only against the blood stages of the malaria parasite (namely trophozoites and schizonts), in which the parasite is actively degrading hemoglobin, but it is not active against infectious sporozoites, liver-stage schizonts and hypnozoites [8, 11]. Previous studies have shown the association of membrane proteins, which are involved in transport of drugs, lipids, and peptides with drug resistance [12–14].

There have been many reports on CQ-resistance from different regions of the world [15–19] including Brazil [20–23]. The widespread emergence and spread of CQ-resistance in *P*. *vivax* (PvCR) represent one of the greatest threats to control and elimination efforts [24]. Moreover, molecular mechanisms of CQ-resistance in *P*. *vivax* are poorly understood and currently there is no validated biomarker for this parasite phenotype, preventing reliable drug resistance surveillance in endemic regions [25].

In the western Brazilian Amazon, in an analysis of 135 individuals, a higher initial mean parasitemia was associated with CQ-resistance in *P*. *vivax* (PvCR) in 5.2% of the patients at day 28. Hemoglobin levels were similar at the beginning of the follow-up period but were significantly lower at days 3 and 7 post-treatment in the patients with resistant infections [22]. Parasites from patients with PvCR presented up to 6.1 and 2.4 fold increase in *pvcrt-o* and *pvmdr-1* expression levels, respectively, compared to the susceptible group [26]. *In vivo* overexpression of both genes, irrespective of the absence of mutations in *P*. *vivax* genes for transporters and folate pathway *P*. *falciparum* ortholog genes linked to CQ-resistance [22, 26], indicates that components of epigenetic regulation may be involved in the PvCR phenomenon, including the effects of nutritional, metabolic and immune factors, as suggested from longitudinal drug resistance studies in complicated and non-complicated malaria [26, 27]. Several studies based on *P*. *vivax* isolates from Southeast Asia have shown the involvement of copy number variation of *pvcrt* or *pvmdr* genes with the CQ-resistant phenotype [28, 29]. In the Brazilian Amazon, copy number amplification of these genes is expected to be very low among *P*. *vivax* strains (0.9%) [30] and below the estimates of CQ-resistance in this area [21]. This suggests an alternative mechanism for CQ-resistance instead copy number amplification.

Metabolomics in malaria, namely targeted metabolomics, was applied almost exclusively to identify *P*. *falciparum* stage-specific changes in metabolic pathways involved in parasite differentiation and invasion in order to better inform drug discovery and design [31–35] or to predict disease severity [36–39]. In this context, metabolomics has been used to gain understanding of the intraerythrocytic development cycle of *P*. *falciparum* in cell culture studies, expanding the knowledge of amino acid and lipid metabolism [40, 41]. Application of untargeted high-resolution metabolomics (HRM) [42] using liquid chromatography coupled to ultra-HRM spectrometry was used with advanced data extraction algorithms [43, 44] and a metabolome-wide association study (MWAS) to identify metabolites associated with *P*. *falciparum* infection in *in vitro* culture samples [45]. The study of the *P*. *falciparum* intraerythrocytic development cycle revealed increased 3-methylindole, a mosquito attractant; succinylacetone, a heme biosynthesis inhibitor; S-methyl-L-thiocitrulline, a nitric oxide synthase inhibitor; and O-arachidonoyl glycidol, a fatty acid amide hydrolase inhibitor [45]. Each of these could be mechanistically important in the parasite’s life cycle and disease manifestations. Coupled with the mechanistic data for metabolic pathways involved in parasite differentiation and invasion, metabolomics results highlight an important need and opportunity to apply HRM for studies of drugs on parasite metabolism and resistance to antimalarials.

Metabolomics remains a relatively new field for malaria research and insights are currently lacking on the metabolic changes that occur during *P*. *vivax* development. In the present study, we examined plasma metabolomics of samples obtained before CQ treatment from patients infected with *P*. *vivax* who were subsequently classified as CQ-R and CQ sensitive (CQ-S) after a 28-day follow-up period in the Brazilian Amazon. A metabolome-wide association study (MWAS) was performed to determine differentially expressed metabolites and perturbed metabolic pathways between CQ-R and CQ-S patients. Results could facilitate development of optimal treatment therapies and clinical diagnostic tests for tracking and therapeutically targeting such processes [46].

## Methods

**Fig 1:**
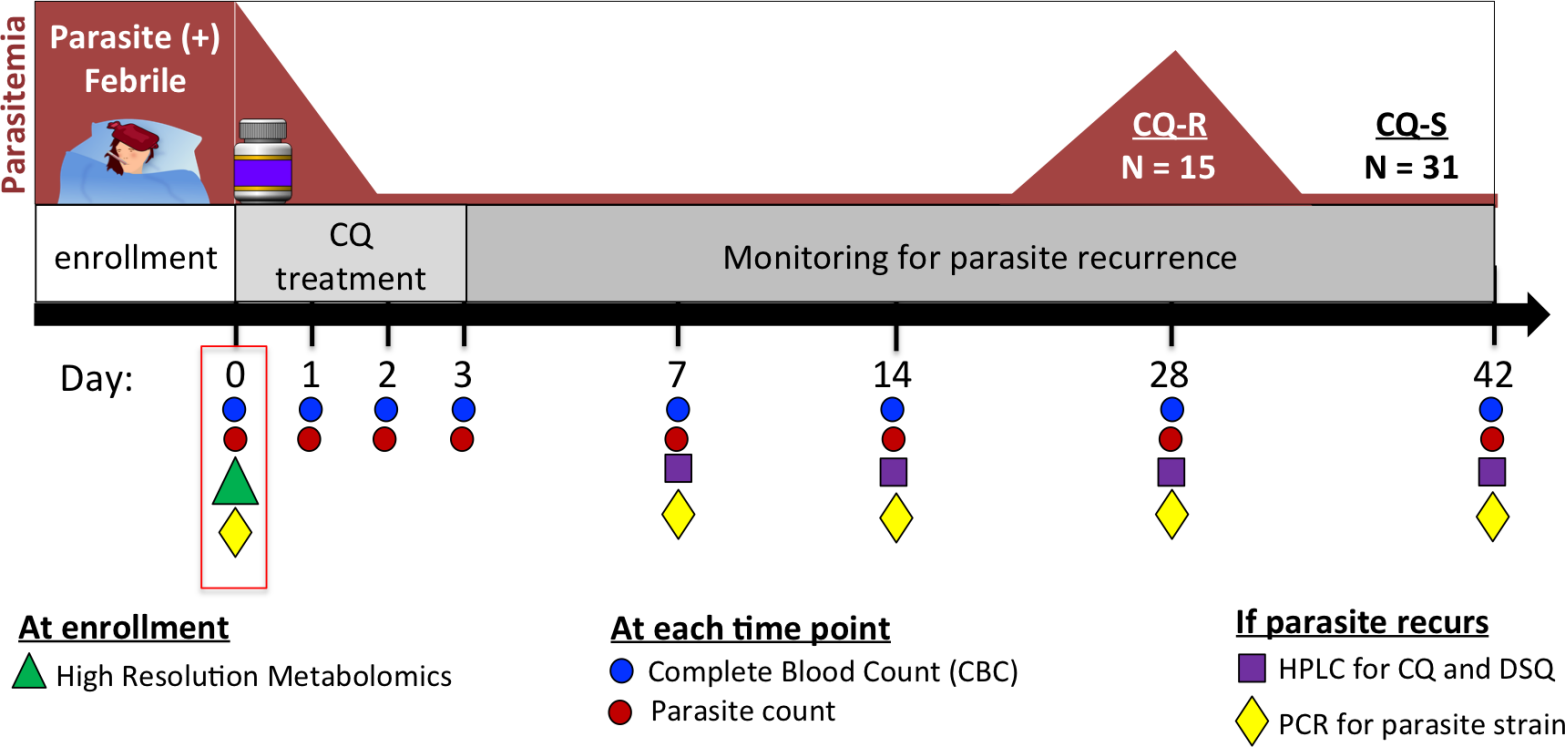
Study design for identifying host metabolite factors that are associated with chloroquine resistance. Schematic depicts timeline of sample collection and processing. Individuals with *P*. *vivax* malaria were enrolled at Day 0 and followed for 42 days. At enrollment, plasma was collected and stored for later processing by high-resolution metabolomics, and parasite strain was determined. Individuals then treated with chloroquine for three consecutive days and were monitored at Days 0, 1, 2, 3, 7, 14, 28 and 42 with complete blood count (CBC) and parasite count to determine if parasites recurred following CQ-treatment. CQ-Resistance (CQ-R) was assessed based on presence of the same strain parasite during the recurrence, along with high levels of CQ and DSQ in the bloodstream, as assessed by HPLC. Metabolomics analysis was performed to compare differences in host metabolites prior to CQ-treatment, comparing individuals who developed CQ-R (N = 15) versus CQ-Sensitive (CQ-S) who did not (N = 31).

Patients with vivax malaria were enrolled in this study from June 2011 to December 2012 at the *Fundacão de Medicina Tropical Doutor Heitor Vieira Dourado* (FMT-HVD), an infectious disease referral center located in Manaus, Western Brazilian Amazon. This study, which required a 42-day follow-up period, was approved by the FMT-HVD Institutional Review Board and the Brazilian National Ethics Committee (CONEP) (IRB approval #: CAAE: 12516713.8.0000.0005). All protocols and documentation were reviewed and sample shipments approved by the Emory IRB. A written informed consent was obtained from study participants. In case of children/minors, a written informed consent was obtained from parents and legal guardians on behalf of their participants. A schematic of the study design is shown in Fig 1. Male and female patients were eligible for inclusion if aged 6 months to 60 years, bodyweight ≥5 kg, presenting a blood parasite density from 250 to 100,000 parasites/microliter and axillary temperature ≥37.5°C or history of fever in the last 48 hours. Exclusion criteria were: use of antimalarials in the previous 30 days, refusal to be followed up for 42 days and any clinical complication. Patients received supervised treatment with 25 mg/kg of CQ phosphate over a 3-day period (10 mg/kg on day 0 and 7.5 mg/kg on days 1 and 2) according to the guidelines of the Brazilian Ministry of Health. Primaquine (0.5 mg/kg per day for 7 days) was prescribed at the end of the 42-day follow-up period [47]. Patients who vomited the first dose within 30 minutes after drug ingestion were re-treated with the same dose. Patients were evaluated on days 0, 1, 2, 3, 7, 14, 28 and 42 and, if they felt ill, at any time during the study period. Blood smear readings, complete blood counts, and diagnostic polymerase chain reaction (PCR) amplifications were performed at all time points. Three aliquots of 100 μL of whole blood from the day of a recurrence were spotted onto filter paper for later analysis by high performance liquid chromatography (HPLC) to estimate the levels of CQ and desethylchloroquine (DCQ) as previously described [19]. In this study, CQ-resistance with parasitological failure was defined as parasite recurrence in the presence of plasma concentrations of CQ and DSQ higher than 100 ng/mL and microsatellite analysis revealing the presence of the same clonal nature at diagnosis and recurrence [26]. The CQ-sensitive control group consisted of patients with no parasitemia recurring during follow-up period. A group of 20 healthy individuals from Brazil was used as non-malarial control group. All samples were processed after blood collection and immediately the plasma was separated and further stored at -80°C until metabolomics.

### *Plasmodium vivax* malaria diagnosis

Thick blood smears were prepared as recommended by the Walker technique and evaluated by an experienced microscopist [48]. Parasite densities (parasites/μL) were calculated by counting the number of parasites per 500 leukocytes in high magnification fields, and the number of leukocytes/μL per patient. In addition, differential counting of asexual forms (ring-stage parasites, mature trophozoites and schizonts) was performed to ensure that there was no difference between groups of cases and controls. Afterwards, real-time PCR was performed to confirm *P*. *vivax* mono-infection. The extraction of total DNA from whole blood was performed using the QIAamp DNA Blood Mini Kit (Qiagen, USA), according to the manufacturer’s protocol. The DNA was amplified in an Applied Biosystems 7500 Fast System using primers and TaqMan fluorescence labeled probes for real time PCR [49].

### High-resolution metabolomics

The composition of the metabolites present in plasma samples was determined using liquid chromatography-HRM spectrometry (LCMS, Accela- LTQ Velos Orbitrap; mass-to-charge, *m/z*, range from 85–2000, positive electrospray ionization). Aliquots of 200 μl of plasma were treated with acetonitrile (2:1) with 14 stable isotope internal standards ([^13^C_6_]-D-glucose, [^15^N]-indole, [2-^15^N]-L-lysine dihydrochloride, [^13^C_5_]-L-glutamic acid, [^13^C_7_]-benzoic acid, [3,4-^13^C_2_] cholesterol, [^15^N]-L-tyrosine, [trimethyl-^13^C_3_]-caffeine, [^15^N_2_]-uracil, [3,3-^13^C_2_]-cystine, [1,2-^13^C_2_]-palmitic acid, [^15^N, ^13^C_5_]-L-methionine, [^15^N]-choline chloride, and 2′- deoxyguanosine-^15^N_2_,^13^C_10_-5′-monophosphate), centrifuged to remove protein, and analyzed in triplicate with a 10 μL injection volume on a C18 reverse phase column with a formic acid/acetonitrile gradient [50]. Several nutrition and health assessment studies and cross-laboratory comparisons have shown that the C18 chromatography can be used for quantification of several endogenous and exogenous metabolites involved in the amino acids metabolism, fatty acid metabolism, nucleotide metabolism, vitamin coenzymes, and environmental chemicals [51, 52]. Although the methods use ultra-high resolution mass spectrometry, the 10-minute gradient used in this study cannot resolve structural isomers and isobaric species [44]. Peak detection, noise removal, and alignment was performed using apLCMS v6.0.1[53] and xcms v1.44[54] with xMSanalyzer v2.0.4[43], yielding a data table with accurate mass (*m/z*), retention time and intensity for each *m/z* feature across all samples. An *m/z* feature is defined as the unique combination of *m/z*, retention time, and intensity profile. Batch-effect correction was performed using ComBat [55].

### Bioinformatics and statistics

Descriptive statistics were employed to evaluate patient characteristics to ensure distributional assumptions for statistical tests were met. A bivariate analysis was performed to identify variables associated with CQ-R using t-test or Wilcoxon Rank-Sum test for continuous variables and Chi-square test for categorical variables. Metabolomics data were filtered to remove features not present in at least 80% of one group or >50% of all samples. After filtering, missing values were imputed by one-half of the lowest signal detected for that feature across all samples [56]. Data were then log_2_ transformed and quantile normalized [57, 58]. Both univariate and multivariate methods were used to identify differentially expressed *m/z* features between CQ-R and CQ-S groups. For univariate analysis, a multiple linear regression model with log_2_ transformed intensities of *m/z* features as dependent variable and response to CQ treatment (CQ-R versus CQ-S) and potential confounding factors (platelet count, age, and gender) as independent variables was fitted for each *m/z* feature. Multiple hypothesis correction was performed using the Benjamini-Hochberg false discovery rate (FDR) correction method [59]. Type 1 (-log_10_ *p* vs *m/z*) and Type 2 (-log_10_ *p* vs retention time) Manhattan plots were used to visualize the pattern of differential expression across all features with respect to molecular mass and chemical properties, respectively. Multivariate analysis was performed using partial least squares discriminant analysis (PLS-DA) implemented in the R package mixOmics v5.2.0, and discriminatory features were selected using the variable importance for projection (VIP>1.5) measure [60]. Fold change of raw intensity values was calculated for each feature as a ratio of average intensity from CQ-R and CQ-S groups. A consensus feature selection framework was used such that only features selected by both univariate and multivariate methods with a fold change greater than two between CQ-R and CQ-S groups were considered as discriminatory features. Two-way hierarchical clustering analysis (HCA) was performed to visualize the relationship between subjects and discriminatory features between CQ-R and CQ-S groups. The classification accuracy of discriminatory features and clinical variables was evaluated using a support vector machine (SVM) classifier and a 10-fold cross-validation procedure, which was repeated ten times. Permutation testing was performed by randomly shuffling the class labels of the subjects. The abundance level of top discriminatory metabolites in subjects with CQ-R and CQ-S parasites was compared to 20 controls from Brazil, pooled plasma from 20 healthy individuals from the United States, and National Institute of Standards and Technology (NIST) pooled Standard Reference Material SRM1950 [61]. 95% confidence intervals were used to represent the metabolite levels for controls, CQ-R, and CQ-S groups.

### Metabolite annotation, pathway analysis, and MS/MS

Annotation of discriminatory features was performed using xMSannotator v1.2 with the Human Metabolome Database (HMDB v3.6) [62]. xMSannotator uses adduct/isotope patterns, correlation in intensities across all samples, retention time difference between adducts/isotopes of a metabolite, and network and pathway associations for associating *m/z* features with known metabolites and categorizing database matches into different confidence levels. This multi-step annotation process reduces the number of false matches as compared to only *m/z* based database search [62]. Mummichog v0.10.3 was used to perform pathway enrichment analysis using *m/z* features that were significant at *p*<0.05 and had VIP>1 [63]. Although the annotation step in mummichog at the individual metabolite level includes false matches, the software uses statistical tests to compare the enrichment pattern of the real metabolite subsets with null distribution, thereby allowing prioritization of pathways for further evaluation [44]. MS/MS analysis of the top discriminatory features with high confidence matches in xMSannotator was performed on a Dionex UHPLC system using C18 chromatography (Higgins Analytical) coupled to a Thermo Fisher Orbitrap Fusion mass spectrometer (HCD—35 eV, 1 AMU isolation window, 120,000 resolution). Raw MS/MS data was processed using DeconMSn [64] and the experimental spectra were compared to MS/MS spectra in Metlin and mzCloud [65, 66].

## Results

Forty-six patients were included in all analyses, 15 CQ-R and 31 CQ-S. The mean age was 33±16.4 years and 10 were female (Table 1). The median hemoglobin at the time of enrollment was 13.75 g/dl (IQR 12.4–14.28 g/dl). The median parasitemia at the time of enrollment was 1.9x10^3^ parasites/μl (IQR 0.8–3.3 x10^3^ parasites/μl). Bivariate analyses were performed to find variables associated with the study outcome and platelet count levels at the time of enrollment were found to be associated with CQ resistance (*p*<0.05, Table 1).

**Table 1:** Demographic and laboratory characteristics of 46 patients with *P*. *vivax* infections assessed for CQ resistance in Manaus, Brazil, 2011–2012.

Variable	Chloroquine resistant (N = 15)	Chloroquine sensitive (N = 31)	P-value
Age (years)	28.3 ± 15.1	35.6 ± 16.8	0.15
Gender			
Male	12 (80%)	24 (77.4%)	1
Female	3 (20%)	7(22.6%)	
Hemoglobin (g/dl)	13.6 IQR [12.4–14.1]	13.8 IQR[12.4–14.4]	0.88
Leukocytes (10^3^ cells/μl)	4.9 IQR [4.7–6.0]	6.4 IQR[5.3–7.4]	0.06
Platelets (10^3^ cells/μl)	86 IQR [54.5–120.5]	130 IQR [98.5–157.5]	0.02
ALT (U/dl)	30 IQR [22.5–54.5]	29 IQR[20.5–38]	0.53
Glucose (mg/dl)	121 IQR [112–130.5]	129 IQR[104.5–161.5]	0.58
Total bilirubin (mg/dl)	0.95 IQR [0.7–1.21]	1.27 IQR[0.79–1.54]	0.25
Creatinine (mg/dl)	0.87 ± 0.32	0.91 ± 0.24	0.68
Parasitemia (10^3^ parasites/μl)	1.3 IQR [0.7–2.6]	2.6 IQR[1.4–3.5]	0.22
Gametocytes	28.2 IQR[0–90.9]	35.8 IQR[4.6–55.6]	1

Abbreviations: IQR = Inter-quartile range. ALT: Alanine transaminase.

P-values obtained using t-tests or Mann-Whitney U test.

**Fig 2:**
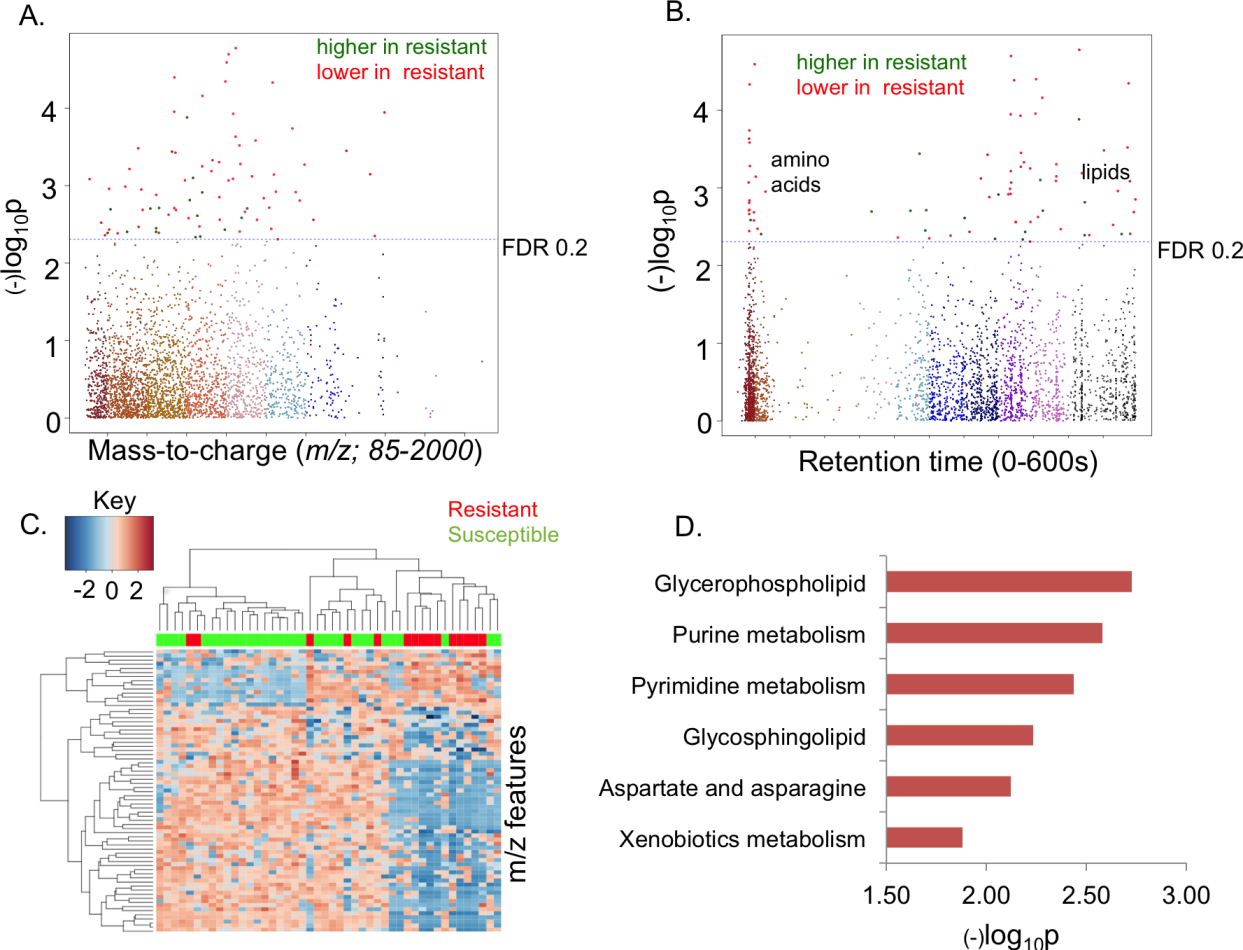
Identification of metabolic features associated with CQ resistance. A) Type 1 Manhattan plot, -log_10_ *p* vs mass-to-charge. 81 *m/*z features with a broad range of *m/z* were found significant at FDR 0.20 threshold. Green dots represent the features that were up-regulated in the CQ-Resistant group and the red dots represent the features that were higher in the CQ-Sensitive group; B) Type 2 Manhattan plot, -log_10_ *p* vs retention time, Majority of features had retention time greater than 4 minutes, which is consistent with elution profile of lipids on a C18 column; C) Two-way hierarchical clustering analysis using discriminatory features; D) Mummichog enriched pathways.

To determine whether metabolic differences occurred between patients subsequently classified as CQ-R and CQ-S, HRM was performed on associated samples collected prior to initiation of CQ treatment (Metabolomics Workbench ID: ST000578). High-resolution metabolomics provided data for 21,360 *m/z* features; after filtering for missing values, 3,049 *m/z* features were log_2_ transformed and quantile normalized for downstream statistical analyses using a consensus feature selection framework (S1 Table). Using the univariate approach, 81 *m/z* features were selected as discriminatory features between CQ-R and CQ-S groups at FDR<0.20 (Fig 2A and 2B). The green (higher in CQ-R) and red (lower in CQ-R) circles above the dotted horizontal line (FDR 0.2 threshold) represent the discriminatory features. The type 1 Manhattan plot shows discriminatory features with a broad range of *m/z*, -log_10_ *P* versus *m/z* (Fig 2A). 77% of the discriminatory features had retention time greater than 4 minutes (Fig 2B). This is consistent with the elution pattern of lipids using reverse-phase chromatography [67]. The less stringent FDR cutoff could facilitate identification of biologically meaningful associations [68]. As an alternative approach, discriminatory features were selected using the Variable Importance for Projection (VIP) scores in a PLS-DA model, which is a multivariate method. Sixty nine of the 81 discriminatory features selected using the univariate approach were also selected at a VIP threshold of 1.5 and had greater than 2-fold increase or decrease in abundance levels between the CQ-R and CQ-S groups (S1 and S2 Tables). Less than 12% of the discriminatory features were associated with the potential confounders (*p*<0.05) in the linear regression model (S2 Table).

Two-way hierarchical clustering analysis (HCA) was performed using the discriminatory features to identify clusters of samples and *m/z* features. Hierarchical clustering analysis showed that the discriminatory features grouped into 14 clusters that could be combined into two major clusters comprised of features with differential expression patterns (up-regulated or down-regulated) between the two groups (Fig 2C). Pathway enrichment analysis using Mummichog showed significant enrichment (*p*<0.05) of several pathways related to lipid and amino acids metabolism: glycerophospholipid metabolism, glycosphingolipid metabolism, aspartate and asparagine metabolism, purine and pyrimidine metabolism, and xenobiotics metabolism (Fig 2D and S3 Table).

Annotation of the top discriminatory features using xMSannotator followed by MS/MS evaluation showed that a subset of features was glycerophosphocholines (S1 Fig and S2 Table). The acquired MS/MS spectra for *m/z* 510.3535 (S1A Fig) matched the spectra for LysoPC(17:0) (level 2 confidence level based on Schymanski et al. [69]) in Metlin. The MS/MS included diagnostic fragments *m/z* 184.0734 (phosphocholine) and *m/z* 104.1071 (choline) for LysoPCs [70]. The MS/MS spectra for *m/z* 516.3058 annotated as M+Na form of LysoPC (16:1) using xMSannotator had one diagnostic fragment, *m/z* 104.1071 (choline) consistent with glycerophosphocholines (S1B Fig; level 2 confidence level based on Schymanski et al. [69]). However, xMSannotator assigned multiple co-eluting and correlated features to different forms (M+H, M+Na, and ^13^C M+H) of LysoPC(16:1). Additionally, we have previously shown that this feature is significantly correlated with choline and other forms of this metabolite [71].

As an alterative data extraction approach, the data were also processed using XCMS [54]. The glycerophosphocholines were significantly different between CQ-R and CQ-S subjects at *p*<0.05 and had VIP>2 using PLS-DA (S2A Fig). Additionally, pathway analysis based on xcms results also showed enrichment of glycerophospholipid metabolism (S2B Fig), thereby indicating that association of glycerophosphocholines with CQ-R is likely to be a real biological phenomenon and not an analytical artifact.

**Fig 3:**
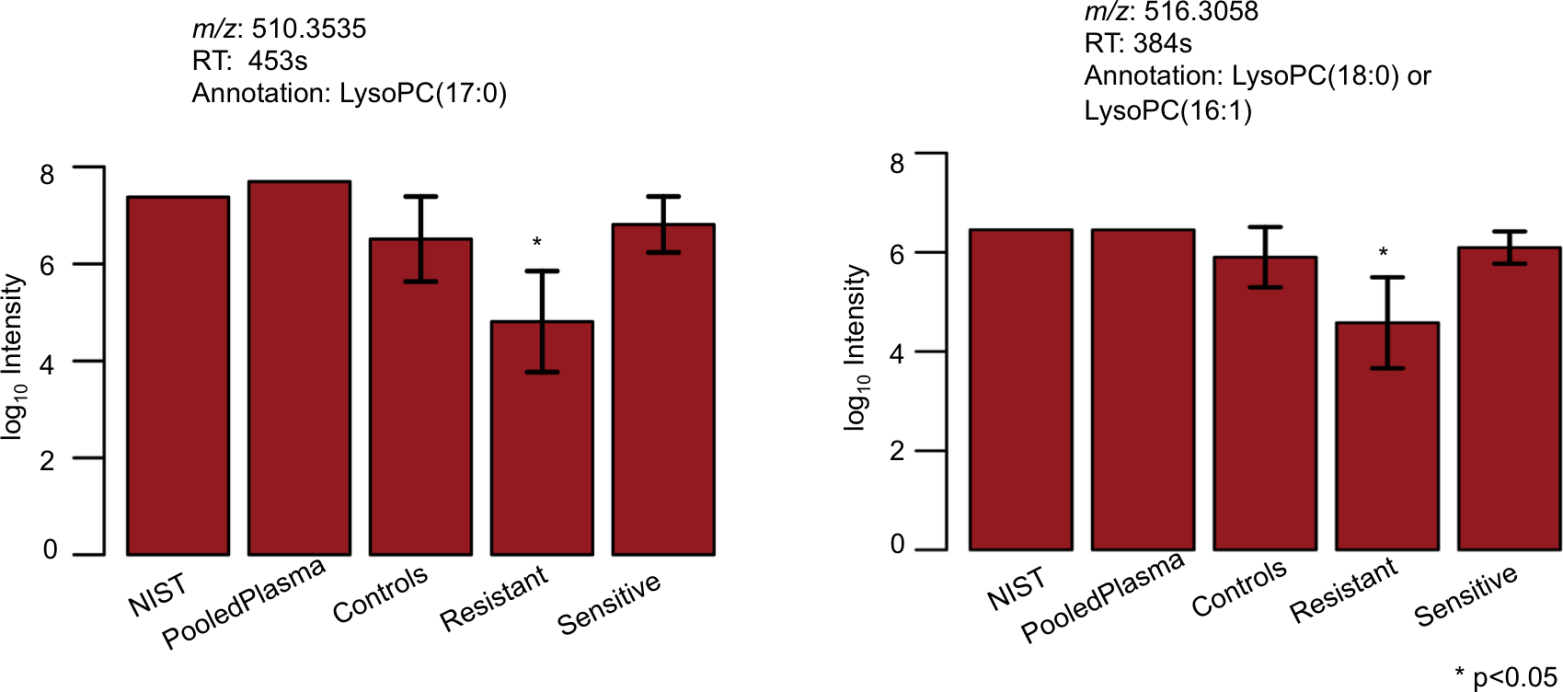
Comparison of glycerophosphocholine abundance levels in NIST, pooled normal plasma (US), healthy controls (Brazil), CQ-Resistant (*P*. *vivax*), and CQ-Sensitive (*P*. *vivax*) groups along with 95% confidence intervals. The glycerophosphocholines were found to be lower in CQ-Resistant group as compared to CQ-Sensitive and other control samples (*p*<0.05).

The abundance levels of the glycerophosphocholines were significantly lower in the CQ-R group as compared to CQ-S subjects and also to independent control samples NIST SRM1950 pooled plasma samples, plasma samples from 20 healthy controls from Brazil, and plasma samples from 20 controls from the US, with *p*<0.05 regarded as significant (Fig 3).

**Fig 4:**
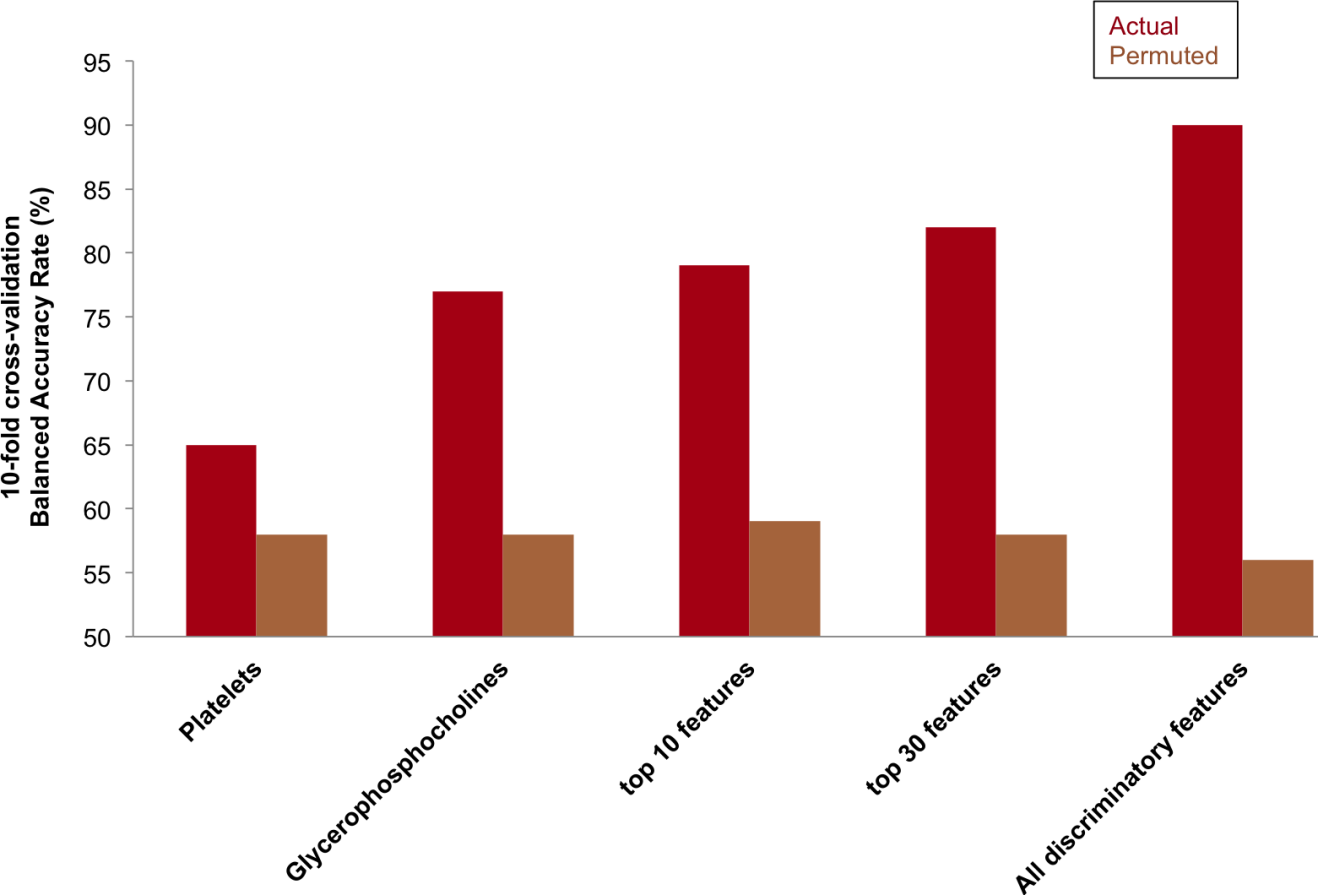
10-fold cross-validation analysis using clinical variables and top discriminatory metabolic features. 10-fold cross-validation classification accuracies varied from 65% to 89.6% using platelet count, glycerophosphocholines, top 10, top 30, and all 69 discriminatory features. The average permuted accuracies (N = 1000 permutations) varied from 55–58%.

Comparisons of 10-fold cross-validation accuracy using only platelet counts, only glycerophosphocholines, top 10 and top 30 discriminatory features ranked based on VIP, and using all 69 discriminatory metabolic features show that the metabolic features allow up to 89.6% 10-fold classification accuracy as compared to 65% using only the platelet counts (Fig 4). Both clinical variables and metabolic features performed better than the randomly generated models.

## Discussion

Here, by comparing the plasma metabolic profiles before CQ treatment of individuals with *P*. *vivax* infections in Brazil, we have identified metabolic signatures that could allow prediction of PvCR. Previous studies did not find any associations between clinical response to CQ and polymorphisms in the *pvcrt-o*, *pvmdr1*, *pvdhfr*, *pvmrp1*, and *pvdhps* genes in *P*. *vivax* subjects [22, 26, 28, 72–75]. The correlation between *ex vivo* CQ resistance and sequence polymorphisms in PvCR candidate genes is limited and contradictory [15, 76]. In terms of gene transcription, however, parasites from patients with PvCR presented up to 6.1 and 2.4 fold increase in *pvcrt-o* and *pvmdr-1* transcription levels, respectively, compared to the susceptible group [26]. Caution is needed when attempting to extrapolate the *ex vivo* biomarkers of resistance to the clinical response, since *pvcrt-o* transcription was not a primary determinant of *ex vivo* drug susceptibility [77]. These previous observations suggest that host-parasite interaction factors, such as nutritional and immune components and clinical severity grading, rather than parasite constitutional factors *per se*, may be involved in PvCR [26, 27, 78]. In this context, coordinated use of metabolic phenotyping of samples from *P*. *vivax* patients presenting with well-defined clinical resistance to CQ holds much promise for the development of new tools to understand the biological process of PvCR and to identify potential biomarkers of PvCR.

Chloroquine mechanism(s) of action has been an intense area of research for decades. Evidence supports that the principal target is the heme detoxification pathway in the digestive vacuole, where the parasite degrades erythrocytic hemoglobin and polymerizes the liberated toxic heme monomers to inert biocrystals of hemozoin [8, 9]. Inhibition of heme polymerization would lead to a toxic milieu to the parasite with its own excreta. Thus, *Plasmodium* with a low hemozoin production phenotype should present as CQ-R [79] as observed for *P*. *falciparum* [80]. Catabolism of host hemoglobin in *Plasmodium berghei*-infected reticulocytes is also down regulated in CQ-resistant parasites [81]. Interestingly, replication of *Plasmodium* developing inside reticulocytes, such as *P*. *vivax*, can occur without hemozoin formation, resulting also in CQ-resistance [82]. In the current study, lipid (glycerophospholipid and glycosphingolipid metabolism) and amino acids (aspartate and asparagine metabolism) pathways were dissimilarly expressed in PvCR carriers. Lipid membranes and proteins are typically involved in biomineralization processes in *Plasmodium* [83–87], suggesting a modulation in terms of quantitative expression of these groups of metabolites in *P*. *vivax* with different degrees of CQ sensitivity.

Here, glycerophospholipids and glycosphingolipids metabolism pathways were differentially expressed in CQ-R and CQ-S subjects before treatment. Glycerophospholipids are the main *Plasmodium* membrane constituents, with the predominant phosphatidylcholine and phosphatidylethanolamine lipids originating from the parasite-encoded enzymatic machinery for membrane neogenesis, which requires high amounts of phospholipids [88]. Previous studies have shown alterations in phospholipase A2 (PLA2) activity, which is involved in the glycerophospholipid metabolism, during *P*. *vivax* malaria in human studies and in erythrocytes infected with *P*. *falciparum* following CQ treatment [89]. Chloroquine has high affinity for membrane phospholipids, and inhibition of *Plasmodium* PLA2 may be important for therapeutic action [89, 90]. Furthermore, previous studies have shown the involvement of transporter genes and membrane proteins that involved in the transport of drugs and lipids with antimalarial drug resistance [14]. Glycosphingolipids are important components of cellular membranes involved in various biological functions, and their biosynthesis was described in *Plasmodium* by an active malarial glucosylceramide synthase [91, 92]. Marked lower levels of glycerophosphocholines in CQ-R compared to CQ-S and control groups and perturbation of the glycerophospholipid metabolism based on the pathway analysis indicates that there could be perturbed PLA2 activity in the CQ-R subjects before treatment. Alternatively, CQ-R parasites may have the ability to utilize the host glycerophosphocholines, and therefore be able to survive even when CQ inhibits the *Plasmodium* PLA2. There is a well-documented link between hemozoin formation and lipid membrane metabolism [93–96]. Considering that host hemoglobin catabolism and hemozoin production are reduced in CQ-R infected cells [79], a differential expression in glycerophospholipids, glycosphingolipids and glycerophosphocholines pathways are expected from parasites, with further decreased levels of their metabolites. Since glycerophosphocholines levels were decreased in mice presenting severe malaria, especially cerebral malaria [97, 98], one also speculates that parasite virulence or host-parasite interactions may be different in CQ-R and CQ-S phenotypes. In addition, a slower hemoglobin digestion process in patients with CQ-R infected cells may result in a milder inflammatory profile and in a reduced cell turnover of those metabolites compared to CQ-S carriers [99, 100].

In addition to pathways related to lipid metabolism, metabolomics also suggests perturbations in aspartate and asparagine metabolism and nucleotide metabolism. *Plasmodium* has a rudimentary pathway for amino acid biosynthesis, depending mainly on host hemoglobin degradation and extracellular sources to meet its amino acid requirements [101]. Of these amino acids, asparagine plays a pivotal role in the parasite life cycle by serving as one of the most abundant amino acids in *P*. *vivax* [102]. Consequently, malaria parasites have retained asparagine synthetase, which catalyzes the formation of asparagine from aspartate [101]. In situations of high parasite load or low-hemozoin producer phenotypes, in which arginine requirements are expected to be higher [103], depletion of blood asparagine levels and increased transcription of parasite asparagine synthetase may occur [104]. *Plasmodium* parasites are unable to synthesize purines *de novo* and have to salvage them from the host through endogenous host erythrocyte transporters [105]. As observed for amino acids, dissimilar requirements of nucleotide by CQ-R and CQ-S phenotypes may explain the higher uptake of purines from the host, which is consistent with our results. Moreover, purine and pyrimidine metabolism pathways have previously been associated with inflammation and enhanced immune cell turnovers [106]. Accordingly, circulating nucleic acids increase in patients with *P*. *vivax* [107], pointing to the involvement of host response leading to differences in nucleotide pathways between groups. Interestingly, metabolomics demonstrates that *Plasmodium* can utilize elements of the reserves of reticulocytes, namely nucleotides, which are absent in mature red blood cells [108]. Phenotype-specific differences in reticulocyte stages tropism or dissimilar trends in differentiation in reticulocyte resident parasites may result in notable differences in the necessity for parasite intrinsic metabolism. The xenobiotic metabolism pathway included two hydroxynaphthalene metabolites (common air pollutants) [109] dependent upon cytochrome P450 activities. Since CQ metabolism is also associated with cytochrome P450 [110], naphthalene and other environmental chemicals could influence this result.

One of the limitations of the current study is the small number of human plasma samples available for comparison. Although a consensus feature selection framework was used with both univariate and multivariate methods to reduce the risk of over-fitting, additional validation studies will be required to replicate these findings in independent cohorts. Further, the host inflammatory state was not extensively explored in this study, and may also influence the results, as plasma metabolic changes are observed during immune responses [111]. More detailed investigations exploring the host immune response alongside the parasite will enable a clearer understanding of the respective roles of host and parasite in the altered metabolic state of the CQ-R individuals. Future work will focus on validating these findings in an independent set of samples, including samples from different endemic regions. Additionally, future investigations will focus on improving our understanding of the CQ-host-vivax relationship.

Host nutritional status may influence malaria susceptibility and host and parasite metabolomics, but the direct effect of a subject’s nutritional status was not assessed in this work when adjusting the analysis. Indeed, evidence of an exacerbating role of malnutrition on malaria can be seen in longitudinal drug resistance studies. A slower parasite clearance, higher parasitemia at presentation and more severe drug resistance were seen in malnourished Rwandan refugees [112]. Likewise, in the Solomon Islands [113, 114] and Malawi [115] malnourished children were significantly more prone to experience treatment failures than those better nourished. These findings emphasize the complex metabolic pathways through which nutrients may influence malaria parasites and host morbidity and bring new insights to explore the previous associations between CQ-resistance and malaria severity [22, 26] using high-resolution metabolomics to integrate nutrition to host and parasite metabolism in the future.

## Conclusion

We present the first report of the use of high-resolution metabolomics to identify metabolites and metabolic pathways related to PvCR. The results show differences in glycerophospholipid and glycosphingolipid metabolism, aspartate and asparagine metabolism, and purine and pyrimidine metabolism pathways in CQ-R vs CQ-S subjects prior to antimalarial treatment. Based on previous studies, low catabolism of host hemoglobin with further lower hemozoin formation in *P*. *vivax*-infected reticulocytes in CQ-resistant parasites could be involved in this sequence of metabolic alteration. Although the number of samples in this study was small, the results demonstrate the future potential of HRM in identifying *P*. *vivax* infected individuals that are likely to show CR, and thus facilitate the design of optimal treatment plans. We present differentially expressed metabolites and perturbed pathways that will require further validation in clinical human cohorts and animal studies. Components of host metabolism regulation may be involved in the PvCR phenomenon, including the effects of nutritional, metabolic and immune factors.

## Supporting information

S1 Table Data table for 3,049 *m/z* features along with metadata and statistical evaluation results. (XLSX)

S2 Table Statistical evaluation and annotation results of 69 discriminatory features. (XLSX)

S3 Table Data for metabolites involved in perturbed pathways shown in Fig 1D. (XLSX)

S1 Fig MS/MS evaluation of *m/z* features matching LysoPCs, A) Comparison of experimental MS/MS spectra for *m/z* 510.3535 annotated as LysoPC (17:0) with reference spectra in Metlin; B) MS/MS fragments for *m/z* 516.3058 annotated as LysoPC(16:1).
(TIF)

S2 Fig Summary of statistical and pathway analysis results based on feature table generated using XCMS. A) Box plots of LysoPC (17:0) and LysoPC(16:1) with *p*<0.05 and VIP>2; B) Pathway analysis results of significant features showed enrichment of glycerophospholipid metabolism pathway.
(TIF)
