# Supplementary figures and images for: Plasma metabolomics reveals membrane lipids, aspartate/asparagine and nucleotide metabolism pathway differences associated with chloroquine resistance in Plasmodium vivax malaria
Source: PLoS One. 2017 Aug 16;12(8):e0182819. doi: 10.1371/journal.pone.0182819 (PMC5559093; doi:10.1371/journal.pone.0182819)

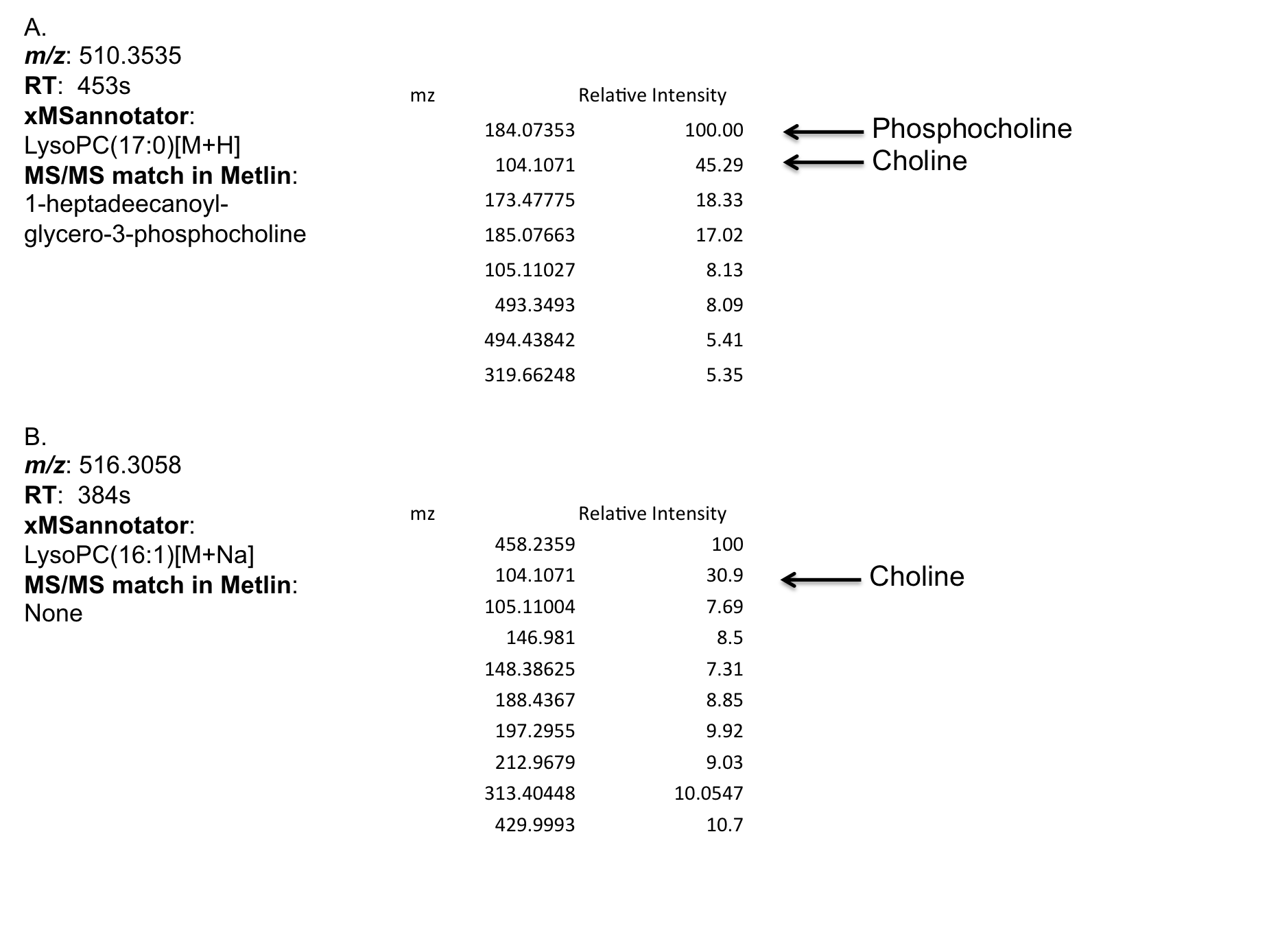

Supplement: S1 Fig — MS/MS evaluation of m/z features matching LysoPCs, A) Comparison of experimental MS/MS spectra for m/z 510.3535 annotated as LysoPC (17:0) with reference spectra in Metlin; B) MS/MS fragments for m/z 516.3058 annotated as LysoPC(16:1). (TIF) [file pone.0182819.s004.tif]

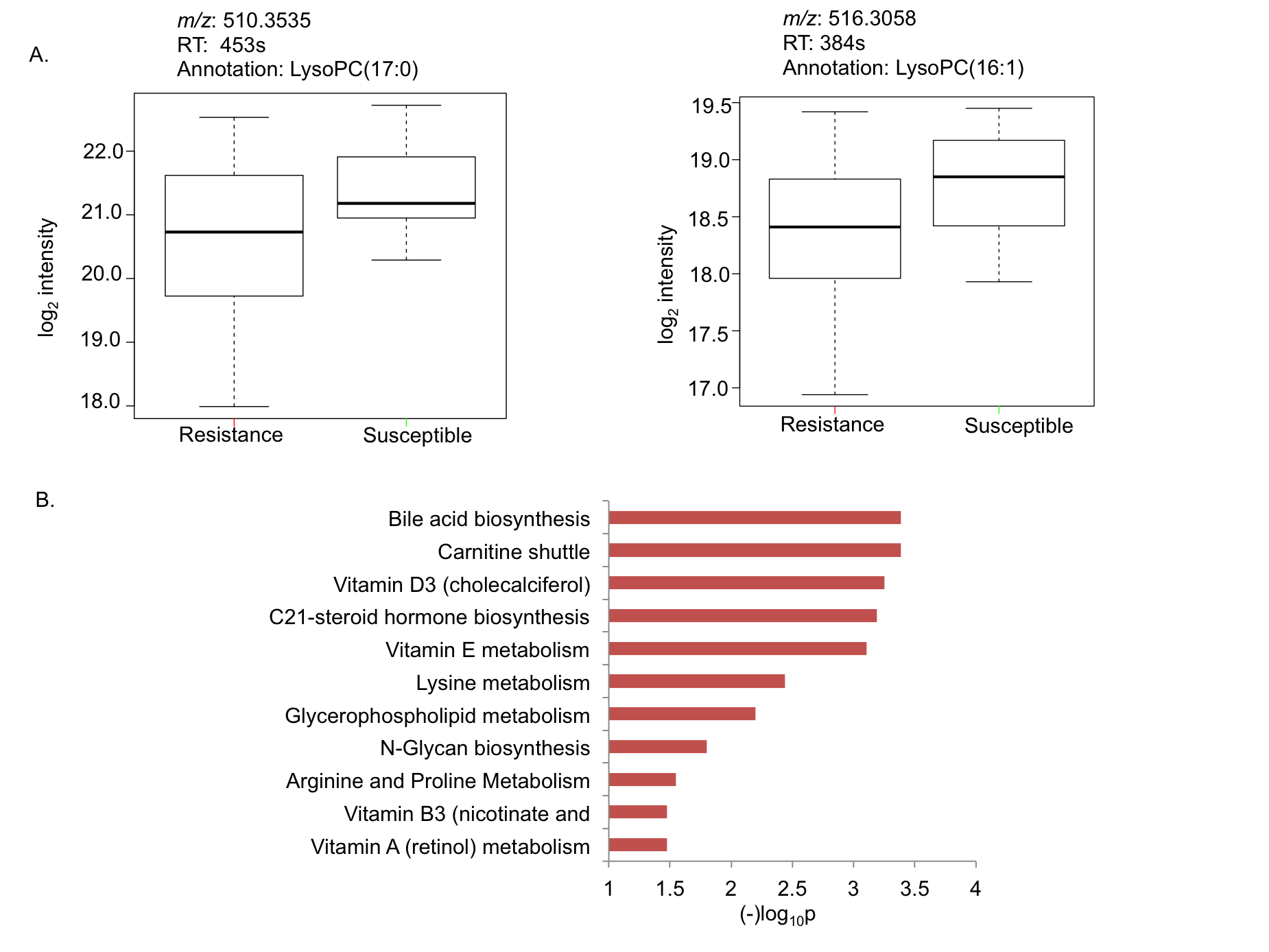

Supplement: S2 Fig — A) Box plots of LysoPC (17:0) and LysoPC(16:1) with p<0.05 and VIP>2; B) Pathway analysis results of significant features showed enrichment of glycerophospholipid metabolism pathway. (TIF) [file pone.0182819.s005.tif]
